# Evaluation of malignant parathyroid tumours in two European cohorts of patients with sporadic primary hyperparathyroidism

**DOI:** 10.1007/s00423-015-1361-4

**Published:** 2015-12-11

**Authors:** Arturs Ozolins, Zenons Narbuts, Andrejs Vanags, Zane Simtniece, Zane Visnevska, Aycan Akca, Denis Wirowski, Janis Gardovskis, Ilze Strumfa, Peter E. Goretzki

**Affiliations:** 1 Department of Surgery, Riga Stradins University, Dzirciema Street 16, LV-1007 Riga, Latvia; 2 Department of Pathology, Riga Stradins University, Riga, Latvia; 3 Department of Surgery, Lukas Hospital, Neuss, Germany

**Keywords:** Parathyroid carcinoma, Primary hyperparathyroidism, Immunohistochemistry, Ki-67, Parafibromin

## Abstract

**Purpose:**

Parathyroid carcinoma (PC) is remarkable for its rare occurrence and challenging diagnostics. PC accounts for 0.1–5 % cases of primary hyperparathyroidism (PHPT). The differentiation from benign tumours is difficult even by morphological criteria. To address these issues, we assessed the PC frequency in two separate European PHPT cohorts and evaluated the demographic, clinical, morphological and molecular background.

**Methods:**

A retrospective study was carried out, using continuously maintained database (2005–2014) of PHPT patients from two tertiary referral university hospitals in Europe. The demographic, clinical data and frequency of PC among surgically treated PHPT was detected. Immunohistochemistry (IHC) was performed to detect parafibromin, representing protein product of *HRPT2* gene and proliferation marker Ki-67.

**Results:**

Both PHPT cohorts were characterised by close mean age values (58.6 and 58.0 years) and female predominance. The frequency of PC differed significantly between the cohorts: 2.1 vs. 0.3 %; *p* = 0.004. PC was characterised by invariable complete loss of parafibromin contrasting with parathyroid adenomas. The proliferation fraction was similar in both PC cohorts (10.6 and 11.0 %). PC showed significantly higher proliferation fraction than typical parathyroid adenomas (1.6 %), atypical adenomas (1.6 %) or adenomas featuring focal loss of parafibromin (2.2 %).

**Conclusions:**

PC frequency can range significantly between the two European cohorts. The differences can be attributable to selection bias of patients referred for surgery and are not caused by discordant definition of malignant parathyroid histology. Diffuse loss of parafibromin and increased proliferation fraction by Ki-67 are valuable adjuncts in PC diagnostics due to significant differences with various clinical and morphological subtypes of adenoma.

## Introduction

Primary hyperparathyroidism (PHPT) is among the most common endocrine diseases. It usually results from a single parathyroid adenoma (PA). Less frequently, tumours can develop in multiple parathyroid glands [1]. Parathyroid carcinoma (PC) is the least common endocrine malignancy representing only 0.005 % of all cancers [2]. PC accounts for 0.1–5 % of all PHPT in contrary to benign PA causing approximately 85 % of PHPT cases [3]. Around half of PC cases are diagnosed at a mean age of 50 years with no sex or race predilection [4]. Most of the patients present with clinical and biochemical manifestations of severe hyperparathyroidism, including severe hypercalcemia, elevated parathyroid hormone (PTH) level as well as renal and osseous complications [5]. A palpable neck mass is present in 30–76 % of patients [4]. However, the diagnosis of PC is rarely made preoperatively.

Aetiology of PC is largely unknown but history of neck irradiation is a known risk factor for all neck cancers. There is no evidence that PC arises from pre-existing benign parathyroid lesions [4]. PC can occur sporadically and in 15 % of cases in association with familial isolated primary hyperparathyroidism and hyperparathyroidism-jaw tumour syndrome (HPT-JT). This is not the case for other syndromes like multiple endocrine neoplasia (MEN1, MEN2A) [3, 6].

Intraoperatively, malignant nature is suspected by the features of local invasion, as well as enlarged and firm to hard affected gland with greyish white cut surface. The mean tumour size reaches approximately 3 cm (range, 1–7 cm), and the average weight ranges around 3 g. However, tumours as large as 67 g have been reported [5, 7]. The diagnosis of PC must be confirmed by the World Health Organisation (WHO) morphological criteria. Extensive invasion of adjacent tissues and metastatic spread represent the two absolute diagnostic criteria [8]. The other criteria include focal coagulation necrosis, irregular dense fibrosis and capsular, vascular or neural invasion. PC most frequently invades the ipsilateral thyroid (89 %), skeletal muscles (71 %), recurrent laryngeal nerve (26 %), oesophagus (18 %) as well as trachea in 17 % of PC patients [9]. Since parathyroids can be located inside the thyroid gland, the mere presence of parathyroid tissue within the thyroid is not sufficient for diagnosing parathyroid carcinoma. Regional metastases are present in 15 % of cases. Distal metastases in the lungs and bones are rare [9, 10].

Despite the refined morphological criteria, the diagnosis of PC remains challenging. Over the last decades, physicians, surgeons and pathologists have experienced difficulties in distinguishing PC from benign disease if the patient was lacking overt invasion and/or metastasis [11]. Among advances in the knowledge of the molecular pathogenesis of PC, the identification of the tumour suppressor gene *HRPT2* and the relevant protein, parafibromin have resulted in a valuable diagnostic tool. *HRPT2* mutations result in loss of parafibromin that can be assessed by immunohistochemistry—a widely available and cheap tissue investigation technique [12]. The proliferation marker Ki-67 has also been characterised as a useful tool, since PC usually has greater Ki-67 expression than adenomas [13, 14]. However, both groups overlap; therefore, the current WHO classification guidelines suggest that patients having Ki-67 expression in more than 5 % of parathyroid tumour cells should not be diagnosed with clear-cut cancer but instead should be followed closer due to an increased risk of malignant course [14].

To address the urgent epidemiological and diagnostic issues, we assessed the PC frequency in two separate European PHPT cohorts and evaluated the demographic, clinical, morphological and molecular background.

## Materials and methods

### Study design

The study was performed as a retrospective investigation, using continuously supplemented database of parathyroidectomies. The database was surgeon-maintained at two tertiary referral university hospitals—Pauls Stradins Clinical university hospital, Riga (Latvia) and Lukas hospital, Neuss (Germany). Patients who were diagnosed with PHPT and underwent surgical treatment (2005–2014) were identified within the databases. The inclusion criteria comprised a verified morphological diagnosis of a parathyroid tumour in a surgically removed tissue material. Patients with positive family history of PHPT, secondary or tertiary hyperparathyroidism were excluded from further analysis. Thus, 982 patients were eligible for the study, including 288 patients from Latvia and 694—from Germany. The data on the final diagnosis, age at the time of operation, sex, main clinical symptoms, preoperative serum calcium (Ca) and PTH levels were retrieved. The frequency of PC among surgically treated PHPT was detected and compared between the two European cohorts. Further, data of patients with proven PC were compared to patients who underwent surgery for benign PHPT.

### Surgical pathology evaluation

A retrospective morphological and immunohistochemical investigation of consecutive surgically treated parathyroid tumours was carried out. The pathology data have been obtained via uniform, protocol-based gross and microscopic examination of the parathyroid surgery materials. Grossly, the largest diameter of a mass lesion was detected, among other findings. The tissues were sampled widely for microscopic examination including multiple sections from the tumour capsule and/or grossly involved adjacent thyroid or soft tissues. The samples were routinely fixed in neutral buffered formalin, processed, embedded in paraplast, cut in 3-μm thickness and stained with haematoxylin and eosin. The slides were examined under light microscopy to detect histological tumour type in accordance with the following criteria.

PC was diagnosed either by any one of definitive criteria, or on the basis of at least three additional criteria. The definitive criteria of PC comprised unequivocal evidence of invasive growth involving the surrounding tissues as the thyroid gland, soft tissues or oesophagus; or vascular or perineural invasion; or the presence of regional or distant metastases. The additional criteria included capsular invasion, mitotic activity exceeding five mitoses/ten high power fields, broad fibrotic bands splitting the tumour parenchyma into nodules, coagulative necrosis, diffuse sheet-like monotonous small cells with high nuclear/cytoplasmic ratio, diffuse cellular atypia and widespread nucleolomegaly. If at least three of these features were present, the tumour was diagnosed as carcinoma; otherwise, the diagnosis of atypical PA was issued. PA was diagnosed on the basis of bland non-hyperplastic morphology [15–17].

### Immunohistochemical visualisation and assessment

Immunohistochemistry (IHC) was performed on representative blocks of tumour tissues. For IHC, 3-μm sections were cut on electrostatic glass slides (Histobond, Marienfeld, Germany). After deparaffinisation, antigen retrieval was performed in a microwave oven (3 × 5 min) using a basic (pH 9.0) tris (hydroxymethyl) aminomethane/ethylenediaminetetraacetic acid (Tris/EDTA) buffer (DAKO, Glostrup, Denmark). After the activity of endogenous peroxidase was blocked, the sections were incubated with primary antibodies for 60 min in the magnetic incubation tray at room temperature. To detect the proliferation activity by Ki-67 expression, monoclonal mouse antibody against human antigen, clone MIB-1 (DAKO), was applied at the dilution 1:100. To detect the expression of parafibromin, representing the protein product of *HRPT2* gene, polyclonal rabbit antibody against human antigen (Abcam; code ab84916) was used at the dilution 1:500. The bound primary antibodies were detected by the enzyme-conjugated polymeric visualisation system EnVision (DAKO), linked with horseradish peroxidase using 3,3′-diaminobenzidine as the chromogen (DAKO). Positive and negative quality controls were invariably performed and reacted appropriately. Parafibromin expression was evaluated as a categorical variable. A case was considered positive if unequivocal nuclear reactivity was present in tumour cells; otherwise, the case was treated as negative [18]. To characterise the expression of Ki-67 by mean proliferation fraction, positive nuclei were counted in 400 consecutive neoplastic cells, and the result was expressed as the percentage. Computed-assisted morphometry was applied for quantitative measurements of nuclear positivity, using NIS-Elements (Nikon, Tokyo, Japan) software to analyse images that were obtained by. Eclipse Ci-L microscope (Nikon) in association with DS-Fi2 camera (Nikon).

The immunophenotype was compared among three clinically distinct categories of parathyroid tumours: (1) PC, (2) atypical adenoma possessing atypical morphology but not fulfilling PC criteria and associated with high preoperative Ca level exceeding 2.9 mmol/l and high preoperative PTH exceeding 250 pg/ml and (3) typical PA lacking all the previously listed features.

### Statistical analysis

The statistical evaluation of the data was carried out using the Statistical Package for Social Sciences (SPSS^®^ version 20) software. In addition, CIA software was used to detect the 95 % confidence interval (CI) as described by Altman et al. [19]. Differences were considered statistically significant if *p* < 0.05.

## Results

The database search yielded 982 eligible patients. The demographic data and clinical features of the PHPT cohorts from Pauls Stradins Clinical university hospital, Riga (LAT) and Lukas hospital, Neuss (GER) are shown in Table 1. Regarding age, there was no difference between the two European cohorts. Females were predominantly affected in both groups. However, the female-to-male ratio differed statistically significantly (*p* = 0.006), as it was 7:1 in LAT group vs 4:1 among GER patients.

**Table 1:** Demographic data and clinical features of PHPT patients in two European cohorts (2005–2014)

Feature	LAT	GER	*p*
No. of patients	288	694	
Mean age (years) ± SD	58.6 ± 12.65	58 ± 13.98	NS
Sex (male/female)	35/253	134/560	0.006
Mean preoperative serum calcium (mmol/l) ± SD	2.89 ± 0.34	2.81 ± 0.28	0.01
Mean preoperative serum parathormone (pg/ml) ± SD	324.56 ± 403.93	182.54 ± 166.83	0.001
Nephrolithiasis (%)	30.2	28.2	NS

*LAT* Pauls Stradins Clinical university hospital, Riga, Latvia; *GER* Lukas hospital, Neuss, Germany; *SD* standard deviation; *NS* not significant

Reference ranges: calcium 2.20–2.60 mmol/l; parathormone 17.00–72.00 pg/ml

The mean preoperative Ca and PTH levels were significantly higher in LAT group compared to those in GER (*p* = 0.01; *p* = 0.001, respectively) (Table 1). Preoperative Ca concentration exceeded 2.9 mmol/l in 35.1 % (95 % CI = 29.8–40.8) of LAT cases vs 32.0 % (95 % CI = 28.6–35.6) GER patients. Preoperative PTH level exceeded 250 pg/ml in 28.5 % (95 % CI = 23.6–34.0) of LAT vs 16.0 % (95 % CI = 13.5–18.9) in GER group. Both these findings were present in 17.4 % (95 % CI = 13.4–22.2) of LAT vs 10.5 % (95 % CI = 8.4–13.0) of GER patients.

PC was confirmed by postoperative histology in 6/288 (2.1 %) LAT and 2/694 (0.3 %) GER patients. The difference was statistically significant; *p* = 0.004. There was one male PC patient in LAT group but the remaining five LAT, and both GER patients were females. The demographic data, clinical features and results of IHC staining for Ki-67 and parafibromin are displayed in Table 2. The mean age of PC diagnostics was 52.2 ± 16.1 years in LAT group. Due to the low number of cases, the mean age was not used to characterise the GER PC patients. In LAT cohort, 4/6 PC cases showed elevated preoperative Ca above 2.9 mmol/l in combination with high preoperative PTH >250 pg/ml. The remaining LAT patients had either elevated Ca (1/6) or PTH (1/6). In GER group, the only pathological biochemical laboratory finding was elevated Ca > 2.9 mmol/l in a single patient.

**Table 2:** Demographic data, clinical features and immunohistochemical marker expression in parathyroid carcinoma

	Age	Sex	Calcium (mmol/l)	Parathormone (pg/ml)	Ki-67^a^ (%)	Parafibromin^b^
LAT	45	Male	3.76	1869.2	9.7	neg
	57	Female	4.07	2031.1	8.3	neg
	36	Female	4.20	2331.4	10.3	neg
	66	Female	3.96	2176.3	22.7	neg
	35	Female	3.03	575.2	4.8	neg
	74	Female	3.04	1097.5	5.7	neg
GER	67	Female	3.10	474.4	12.0	neg
	52	Female	3.40	165.3	10.0	neg

*LAT* Pauls Stradins Clinical university hospital, Riga, Latvia; *GER* Lukas hospital, Neuss, Germany; *neg* negative

Reference ranges: calcium 2.20–2.60 mmol/l; parathormone 17.00–72.00 pg/ml

^a^Percentage of positive cells

^b^Expression as a categorical variable

**Fig. 1:**
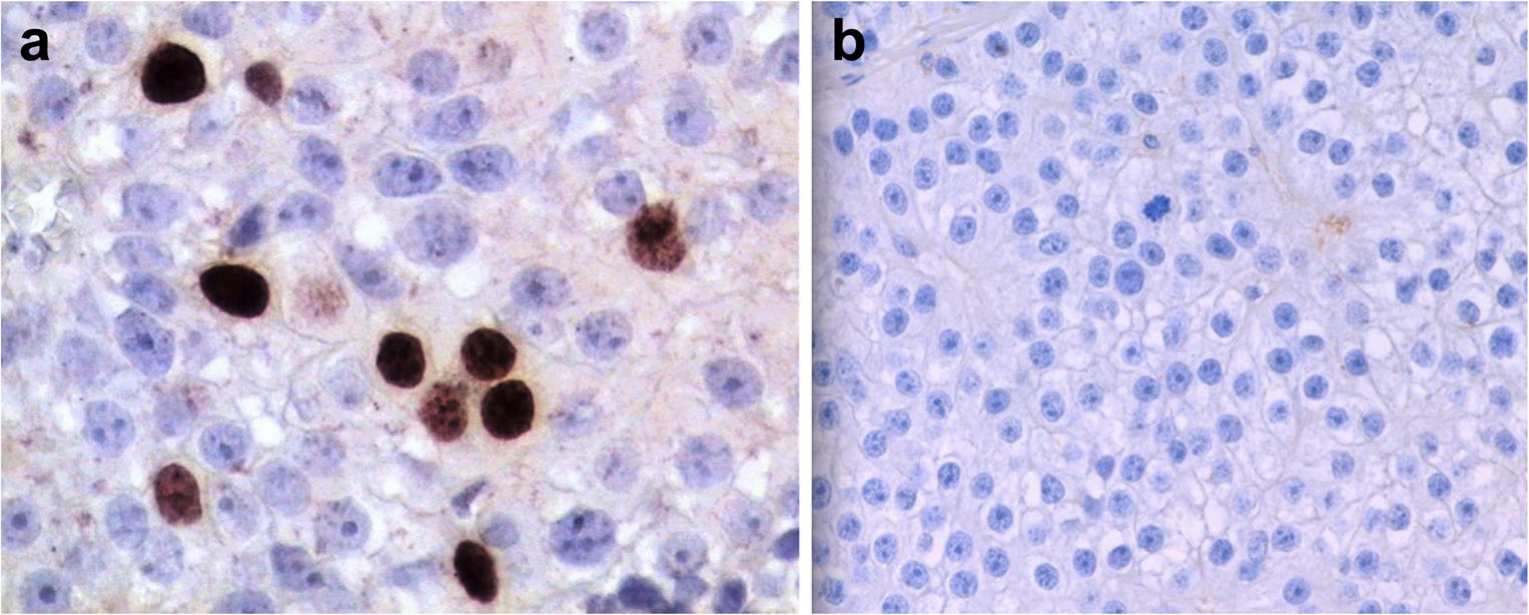
Immunophenotype of the parathyroid carcinoma. **a** High proliferation activity by Ki-67. Immunoperoxidase (IP), anti-Ki-67 (clone MIB-1), original magnification (OM) 400×. **b** Complete absence of parafibromin. Note the presence of mitotic figure and enlarged nucleoli. IP, anti-parafibromin, OM 400×

**Fig. 2:**
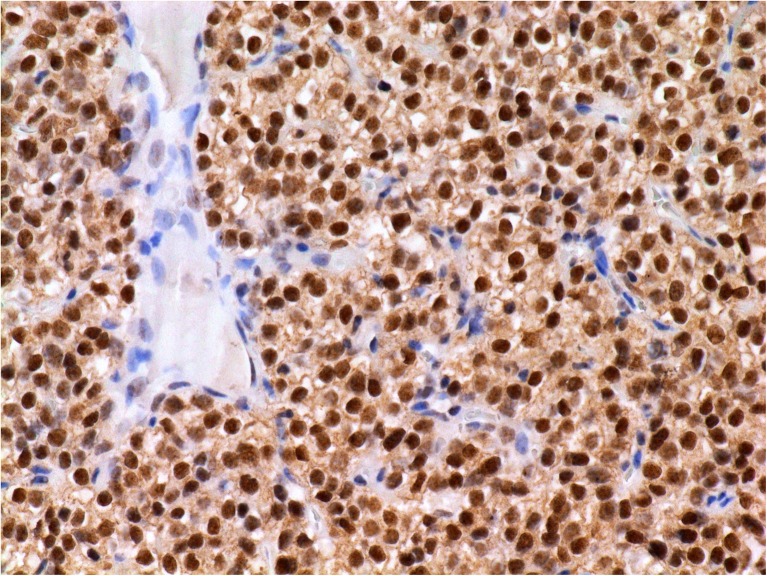
Intense nuclear expression of parafibromin in parathyroid adenoma. Immunoperoxidase, anti-parafibromin, original magnification 400×

In both cohorts, Ki-67 expression was observed in at least 5 % of PC cells (Fig. 1a) except a single case showing proliferation fraction of 4.8 %. The highest proliferative activity was 22.7 %. IHC expression of parafibromin was invariably negative in all PC cases (Fig. 1b) contrasting to mostly positive staining in benign parathyroid tissues (Fig. 2; Table 3).

**Table 3:** Immunophenotype of parathyroid adenomas in relation to distinct clinical features

Target group	Ki-67		Parafibromin	
	Mean ± SD	95 % CI	Frequency (%)	95 % CI
Adenoma (atypical morphology, preoperative Ca > 2.9 mmol/l, preoperative PTH > 250 pg/ml)	1.6 ± 1.00	1.3–2.0	97.5	86.0–100.0
Adenoma (preoperative Ca < 2.9 mmol/l, preoperative PTH < 250 pg/ml)	1.6 ± 1.53	0.7–2.6	100.0	89.1–100.0

*SD* standard deviation, *CI* confidence interval, *Ca* serum calcium level, *PTH* serum parathormone level

Reference ranges: calcium 2.20–2.60 mmol/l; parathormone 17.00–72.00 pg/ml

Further, we analysed the immunophenotype for Ki-67 and parafibromin expression in clinically distinct parathyroid lesions. The mean value of Ki-67 expression in PC was 10.6 % (95 % CI = 4.0–17.1) in LAT group vs 11.0 (95 % CI = 0.0–23.5) in GER patients. The proliferation activity in parathyroid adenomas was statistically and biologically significantly lower, ranging from 1.6 % (95 % CI = 1.3–2.0) to 1.6 % (0.7–2.6) in respect to morphological and biochemical features (Table 3). To estimate the role of tumour heterogeneity regarding parafibromin expression, the proliferation activity by Ki-67 was also compared between PC and PA showing focal loss of parafibromin. However, PA manifesting parafibromin heterogeneity also had significantly lower proliferation activity than carcinomas, namely 2.2 % (95 % CI = 0.6–3.8) vs 10.6 % (95 % CI = 4.0–17.1) considering the larger cohort. The relevant data are shown in Table 4.

**Table 4:** Descriptive statistics of proliferation activity by Ki-67 expression in parathyroid tumours showing diffuse vs focal loss of parafibromin

Target group	Ki-67		Parafibromin
	Mean ± SD	95 % CI	Expression
Parathyroid carcinoma			
LAT (*n* = 6)	10.6 ± 6.23	4.0–17.1	Diffuse loss (6/6)
GER (*n* = 2)	11.0 ± 1.40	0–23.5	Diffuse loss (2/2)
Atypical adenoma + Ca > 2.9 mmol/l and PTH > 250 pg/ml			
LAT (*n* = 7)	2.2 ± 1.70	0.6–3.8	Focal loss (7/7)
GER (*n* = 3)	1.1 ± 0.53	0.6–1.6	Focal loss (3/3)

*SD* standard deviation; *CI* confidence interval; *n* absolute number; *LAT* Pauls Stradins Clinical university hospital, Riga, Latvia; *GER* Lukas hospital, Neuss, Germany

Reference ranges: calcium 2.20–2.60 mmol/l; parathormone 17.00–72.00 pg/ml

## Discussion

Parathyroid carcinoma (PC) is a very rare endocrine entity, but highly aggressive form of PHPT. The frequency of PC among PHPT patients ranges widely between values less than 0.2 % and exceeding 4 %, if the studies from North America and most of Europe are compared to those from Asia and Italy, respectively [20–24]. The rarity of this tumour embarrasses the genetic studies. Thus, it is not well proven, whether PC generally arises de novo or from pre-existing benign PA through the accumulation of genetic abnormalities [25]. There are some case reports describing PC on the background of parathyroid adenoma or hyperplasia, but these data are very scanty [26, 27]. Further, neither imaging nor clinical or laboratory findings can reliably predict PC course in cases lacking any evidence of metastasis, thus impairing both patient’s care and reliability of epidemiological studies [28].

Regarding the PC frequency, significant differences were found between the assessed European PHPT cohorts as PC was diagnosed in 2.1 % LAT but only in 0.3 % GER patients. This could be explained by delayed diagnostics. However, adenoma-to-carcinoma progression has never been shown for PC. It also could be due to geographic variability or by selection bias in the patient referral for surgery. To test these hypotheses, we assessed the demographics, including the age structure, and the molecular characteristics including proliferation activity and loss of parafibromin, suggesting *HRPT2* mutations. In addition, the possibility of discordant morphologic investigation was excluded by strict adherence to the diagnostic criteria described in the Materials and methods.

Historically, delayed presentation of PHPT was characteristic, including functional and structural lesions of the internal organs, nephrolithiasis, hypercalcaemic crisis, muscle weakness, pancreatitis or *osteitis fibrosa cystica* [29, 30]. Nowadays, the full-blown clinical picture has become rare in most PHPT cases but still can be observed in patients with fast growing parathyroid tumours such as PC. The rapid clinical progression of PC in the setting of predominantly clinical diagnostics of PHPT can lead to diagnostics of PC in earlier age than PA, as it was reported in former publications [31]. At present, the majority of patients are diagnosed before the typical PHPT picture occurs, and some may even lack clinical symptoms at the time of diagnosis [32]. The widespread availability of biochemical tests can lead to significantly improved diagnostics in older patients who frequently undergo testing for Ca in association with diagnostics and treatment of other concomitant diseases. Consequently, the epidemiological landscape of PC can now change to the predominance of patients older than 60 years.

We therefore questioned whether this difference in age distribution between PC and PA patients still persists. In our patient cohorts, including 288 LAT and 694 GER patients, no age difference was found (mean age, 58.6 ± 12.65 vs 58 ± 13.98 years). Regarding PC, the age ranged widely (35–4 years), and the mean age was not significantly different from the general PHPT cohorts. Thus, a subfraction of PC patients still is diagnosed in young age influencing the mean age; and aged persons also have been affected. The age analysis also does not support a difference in the time of PHPT diagnosis in the two European cohorts, which otherwise could explain the increased PC frequency among LAT patients. We neither found a difference in sex ratio between PC and PA patients as stated by other authors, who demonstrated a female predominance in PA but rather equal number of females and males in PC cohorts [33]. The predominance of females was shown in all our patient groups. However, higher female-to-male ratio was observed in LAT PHPT patients when compared to PHPT patients from GER (female-to-male ratio, 7:1 vs 4:1).

Altogether, these demographic and clinical data support the independent development of PA and PC contrasting with the tumourigenesis in the other organs such as the large bowel. However, the theory of unrelated genesis of parathyroid tumours has been supported by the assessment of the underlying mutations in PA and PC as well as gene expression by whole genome sequencing [34].

Since a progression of PA to PC has been suggested in some earlier publications [35, 36], higher Ca as well as PTH levels in LAT cohort when compared to GER patients with PHPT, in association with higher PC frequency, could have been interpreted as an evidence of tumour progression. Additional to the previously described age structure and literature-based contradictions to the hypothesis of tumour progression, the experience in MEN1 and MEN2 patients does not demonstrate adenoma-to-carcinoma sequence. The genetic analysis seldom demonstrates MEN1 mutations in PC. As few as ten patients with MEN1 and MEN2 syndromes (seven and three, respectively) have ever been shown to suffer from malignant parathyroid tumours [37]. Thus, PC is surprisingly rare in MEN1 patients, despite the fact that PHPT in MEN1 patients can be detected early and despite the high recurrence rate of PHPT even after effective primary surgery [38].

With increasing numbers of HPT-JT families the number of PC patients will increase. Other sporadic and familial forms of PHPT however will not change the incidence of PC.

Therefore, with exception of PHPT and HPT-JT families, PC occurrence lacks any correlation to occurrence of other patients with sporadic or familial PHPT. This assumption can be generalised and also fits for our LAT and GER patient cohorts. However, germline mutation of *HRPT2* gene is responsible for the autosomal dominant HPT-JT syndrome, manifesting as multiple benign and/or malignant parathyroid neoplasms as well as uterine and renal tumours and ossifying fibromas of the jaw bones [25, 36]. Germline *HRPT2* mutations are also found in patients with familial isolated hyperparathyroidism and in up to 20 % of PC patients who have been primarily classified as sporadic [25], while somatic *HRPT2* mutations are almost exclusively found in up to 77 % of sporadic PC and in less than 1 % of sporadic PA [4, 6, 7].

Parafibromin represents the protein product of *HRPT2*. It is involved in cell transcription, differentiation, proliferation and apoptosis. Although the exact way by which this protein increases the malignant potential of PC remains unclear, it is known to inhibit cyclin D1, which stimulates parathyroid cell growth [12, 39]. In our two European cohorts, all PC patients showed diffuse loss of parafibromin by IHC staining. These data seem surprisingly homogeneous but are in accordance with other publications in this field. Cohorts with higher number of PC patients did present loss of parafibromin staining in approximately 60–100 % [3, 11, 12, 25, 40, 41]. Some of these differences can be attributable to variable definitions of malignant behaviour in parathyroid tumours that can be limited to histologic interpretation only or to clinical follow-up data. In our study, parafibromin expression in so-called atypical PA varied significantly showing either diffuse positivity or focal loss of expression.

A study by Quinn et al. regarding the utility of parafibromin and Ki-67 in the differential diagnostics of parathyroid tumours resulted in the conclusion that loss of parafibromin expression and extent of Ki-67 staining has not been shown to distinguish between PC and atypical adenoma [42]. Those findings are contrary to our data confirming higher proliferation fraction by Ki-67 expression and invariable diffuse loss of parafibromin in PC. Certain other authors also have reported high diagnostic value of parafibromin in the diagnostics of parathyroid carcinoma [15–17]. The study by Witteveen et al. demonstrated a germline and/or somatic *HRPT2* mutation in only four of the 23 PC patients (17 %). These four patients developed a local recurrence and/or distant metastasis during follow-up, suggesting that other factors may also have role in the tumorigenesis of PC [12].

Recently, further studies with new generation genomic sequencing enlightened the genomic landscape of recurring sporadic PC and depicted multiple somatic mutations in other genes than *HRPT2*. Some single nucleotide point mutations were located to well-known genes such as *MTOR*, *MLL2* and *CDKN2C*. Loss of function was found in *PIK3CA* gene, and truncating mutations were demonstrated in *CDKN2C* and *THRAP3* genes. The mutation in *PIK3CA* encoding for a catalytic subunit of PI3K alters the phosphoinositol pathway, while *CDNK2C* mutations impair the inhibition of cyclin-dependent kinases 4 and 6 [43] that stimulate cellular growth. Future genetic investigations have to confirm these data and may lead to specific fingerprints of genetic alterations in PC resulting in molecular classification that will replace the present histology and IHC.

We also analysed Ki-67 expression in benign adenomas concentrating on different preoperative biochemical features including Ca and PTH serum levels. Here, we did not find any statistically significant difference in Ki-67 expression between the subgroups of adenomas (Table 4). The lack of correlation between proliferation activity and the functional tumour manifestations also supports the applicability of Ki-67 IHC in the differential diagnostics of parathyroid tumours.

Since histologic features and clinical behaviour of parathyroid tumours sometimes differ substantially in the follow-up of patients with PC (recurrence and metastasis in histologically seemingly benign tumours as well as long-standing cure of PC by surgery alone), an additional measurement still seems necessary. Whether detection of genetic alteration patterns may fulfil these expectations and will be able to differentiate between benign and malignant parathyroid tumours, at present, is still unknown.

Focusing on the histology as the predictor of clinical course in PC, several morphologic evaluation systems have been reported. Those described by Schantz and Castleman are the most widely used, when PC have to be separated from benign parathyroid tumours [44]. Metastasis and clear-cut invasion remain among the cardinal properties of malignant tumours. The additional morphological features include capsular invasion, presence of trabecular patterns and mitotic figures as well as the presence of fibrous bands. In our study, we also based PC diagnosis on the cardinal tumour signs. However, it still is important to consider the overall clinical picture rather than any single histopathological criteria, when future outcome is prospectively assumed [45].

Thus, significant geographic differences in the PC frequency among the European PHPT patients have shown in the present study. As the age structure and molecular characteristics of PC in both cohorts are similar, biases in patient referral for surgery may be considered as the main underlying cause. Reliable and unified diagnostics should be a cornerstone of the scientific evaluation of rare diseases. The increased proliferation fraction and loss of parafibromin are important molecular features of parathyroid carcinoma that are equally applicable to both tested cohorts. The future studies should elaborate novel morphological and molecular diagnostic tests, considering also the markers explored in the present study.

Limitations of our study are the retrospective analysis of data, lack of epidemiological information concerning the prevalence of PC in Latvian and German population and the low number of patients with PC.

## Conclusions

PC frequency can range significantly between the two European cohorts. The differences can be attributable to selection bias of patients referred for surgery and are not caused by discordant definition of malignant parathyroid histology.

The identification of the parafibromin, encoded by *HRPT2* gene, has provided an important insight in the molecular pathogenesis of PC. Total loss of parafibromin staining and increased proliferation fraction by Ki-67 are valuable adjuncts that may help in differentiating between atypical adenomas and PC.
